# Voltammetry Peak Tracking for Longer-Lasting and Reference-Electrode-Free Electrochemical Biosensors

**DOI:** 10.3390/bios12100782

**Published:** 2022-09-22

**Authors:** Adam McHenry, Mark Friedel, Jason Heikenfeld

**Affiliations:** Department of Biomedical Engineering, University of Cincinnati, Cincinnati, OH 45221, USA

**Keywords:** electrochemical sensor, aptamers, square wave voltammetry, reference electrode, potential drift, peak tracking

## Abstract

Electrochemical aptamer-based sensors offer reagent-free and continuous analyte measurement but often suffer from poor longevity and potential drift even with a robust 3-electrode system. Presented here is a simple, software-enabled approach that tracks the redox-reporter peak in an electrochemical aptamer-based sensor and uses the measurement of redox peak potential to reduce the scanning window to a partial measure of redox-peak-height vs. baseline (~10X reduction in voltage range). This same measurement further creates a virtual reference standard in buffered biofluids such as blood and interstitial fluid, thereby eliminating the effects of potential drift and the need for a reference electrode. The software intelligently tracks voltammogram peak potential via the inflection points of the rising and falling slopes of the measured redox peak. Peak-tracking-derived partial scanning was validated over several days and minimized electrochemically induced signal loss to <5%. Furthermore, the peak-tracking approach was shown to be robust against confounding effects such as fouling. From an applied perspective in creating wearable biosensors, the peak-tracking approach further enables use of a single implanted working electrode, while the counter/reference-electrode may utilize a simple gel-pad electrode on the surface of the skin, compared to implanting working, counter, and reference electrodes conventionally used for stability and reliability but is also costly and invasive. Cumulatively, peak-tracking provides multiple leaps forward required for practical molecular monitoring by extending sensor longevity, eliminating potential drift, simplifying biosensor device construction, and in vivo placement for any redox-mediated sensor that forms parabolic-like data.

## 1. Introduction

Continuous molecular monitoring, beyond glucose, remains an unresolved opportunity with few new technologies coming to market despite the significant need for real-time monitoring of other molecules for disease and drug dose management [1]. The limitations with enzymatic sensors, such as glucose sensors, are that they rely on the enzymatic reaction of their target though specialized enzymes, limiting their ability to be rapidly adapted to sensing other target analytes, and their detection generally to µM–mM concentration ranges [2]. Electrochemical aptamer-based (EAB) sensors represent a potential solution to many of the limitations of enzymatic sensors through modular design for high selectivity and affinity, [3,4] making them adaptable to multiple targets at low concentrations (nM-uM) [5]. The utility of aptamers has been demonstrated for numerous in vivo molecules in real time through electrochemical interrogation of redox-tagged aptamers bound to an electrode [6,7,8,9,10]. However, these devices still have challenges that may limit their widespread application including in vivo device longevity, [11] sensitivity, and the requirement of working, counter, and reference electrodes inserted into the body [12]. Potential drift is minimized by taking measurements relative to the reference for stability. 

**Figure 1:**
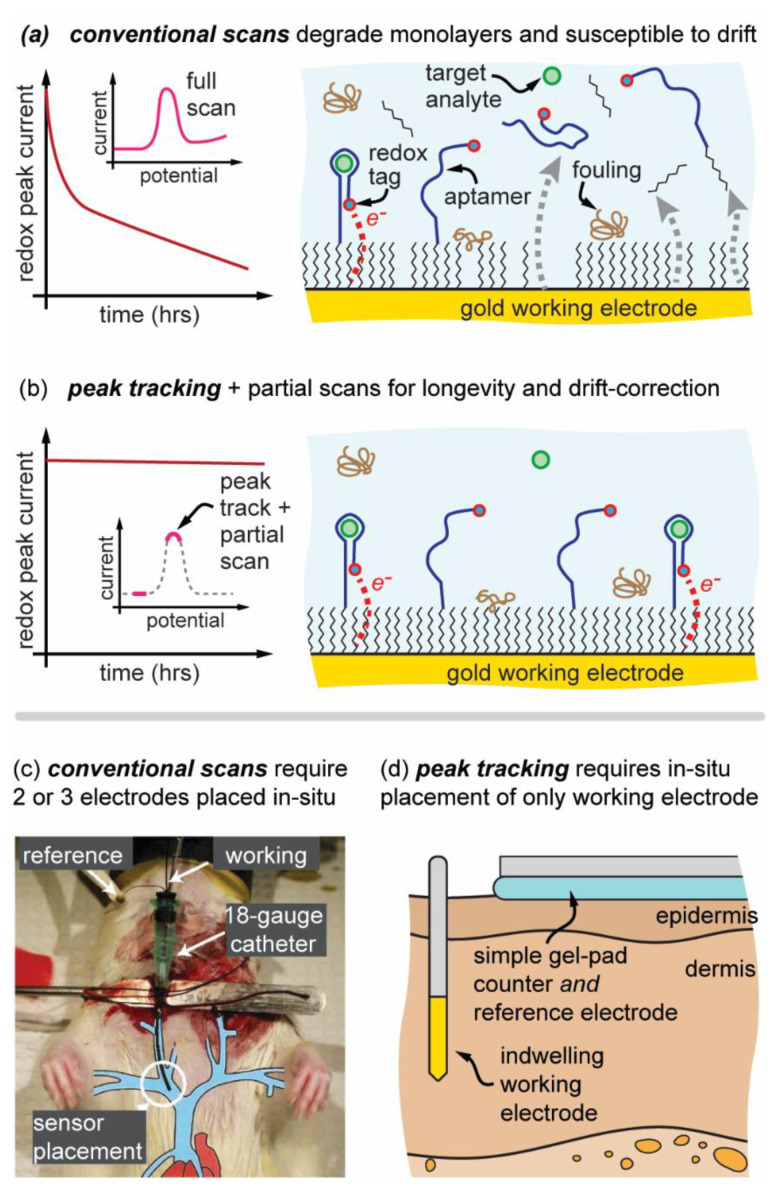
Improvements to traditional sensing techniques through software improvements. (**a**) Conventional E-AB methods susceptible to electrochemically accelerated monolayer desorption and monolayer fouling that can reduce signal and utility whereas (**b**) automatic-peak-tracking improves sensor longevity. (**c**) Conventional in vivo EAB sensing requires more complicated 3-electrode placement inside the body, reproduced from [12] compared to (**d**) auto-peak tracking EAB allows simplified use of a single working electrode in the body.

Presented here is a simple yet powerful automatic-peak-tracking EAB platform [13] that, compared to conventional EABs: (1) dramatically reduces the electrochemical stress applied during measurement and can therefore prolong sensor longevity by preserving the sensor monolayer (Figure 1a,b), [14,15]; (2) eliminates the need for a stable reference electrode not only reducing system complexity from a three-electrode to a two-electrode system, (Figure 1c,d) but also (3) completely resolves long-standing challenges with potential drift in an EAB sensor system [16,17,18]. Importantly, this peak-tracking EAB innovation utilizes simple software controls, allowing adaptation to EAB and other electrochemical sensors with redox-mediated signaling. 

The peak-tracking EAB approach functions in buffered biofluids such as blood or interstitial fluid by leveraging the redox-potential invariability of the methylene blue redox reporter (Figure 1a). The software uses a simple measurement of that redox-potential as real-time method to inform the subsequent range of potential that needs to be applied. The software finds this redox potential using a simple 1st derivative (inflection point) analysis of the measured square-wave voltammogram. Once the redox potential is known, potential drift is tracked and inherently corrected for without a reference electrode. This further can improve sensor longevity, because a full voltammogram does not need to be collected with every measurement, thus reducing the electrical stress placed on the sensor and subsequent degradation (Figure 1b) [14]. Results are demonstrated in vitro using simulated challenges to the peak-tracking EAB sensor including adding serum to induce fouling and potential drift, and testing on porcine skin with simulated ‘motion artifacts’ to demonstrate peak-tracking EABs adaptability and stability. Preliminary results are also shown on improved longevity achieved by reducing the electrical stress placed on the sensor. These peak tracking EAB sensor results are practically important as sensors attempt to progress from the lab and into clinical practice where both longevity and signal stability have been significant challenges, and where simpler in vivo electrode placement is desirable as well.

## 2. Materials and Methods

### 2.1. Materials

Mercaptohexanol (MCH), phosphate buffered saline (PBS), MgCl, Tris(2-carboxyethyl)phosphine hydrochloride (TCEP), serum, and vancomycin were purchased from Sigma-Aldrich (St. Louis, MO, USA). Vancomycin aptamer, [/5ThioMC6-D/CGAGG GTACC GCAAT AGTAC TTATT GTTCG CCTAT TGTGG GTCGG/3MeBlN/] with conjugated methylene blue and thiol ends, was purchased from Integrated DNA Technologies (Coralville, IA, USA) and has been previously characterized. [18] The vancomycin aptamer is often used as a vehicle for wider aptamer research and was therefore an ideal test aptamer for proof-of-concept testing with peak-tracking software (vancomycin detection is not the focus of this paper). Controlled-temperature test enclosures (Happybuy Reptile Incubator 25L) and faradaic shields (TACKMETER WiFi Router Shield, and Electriduct ½” Tinned Copper Metal Braid) were purchased from Amazon (Seattle, CA, USA). A 2 mm gold rod (CHI101), Ag/AgCl reference (CHI111), and Pt counter electrodes (CHI115) were all purchased from CH Instruments (Austin, TX, USA). Polishing pads, and 0.3- and 0.05-micron alumina slurry (ET030) were purchased from eDAQ (Colorado Springs, CO, USA). 

### 2.2. Aptamer Solution Preparation

Aptamer solution was initially created at 100 µM concentration when purchased and was diluted and prepared for use in experimentation as follows. Stock solution was mixed 1:2 with 0.5 M TCEP in DIH_2_O and rested for 1 h at room temperature in the dark. Next, 0.1 M PBS with 2 mM MgCl was used to dilute the aptamer and TCEP to 500 nM for use in sensor functionalization.

### 2.3. Sensor Functionalization

Gold rod electrodes were prepared by polishing in 0.3 and 0.05 µm alumina slurry using a ‘figure eight’ pattern for one minute in each slurry. Next, electrochemical cleaning was performed using cyclic voltammetry from −1 to −1.6 V in 0.5 M NaOH and acid cleaning from 0 to 1.6 V in 0.5 M H_2_SO_4_ by 1 volt per second for 300 cycles with a CHI684 potentiostat. Sensors were rinsed in DI water and functionalized with aptamer by pipetting 25 µL of 500 nM Vancomycin aptamer in PBS on the upward facing gold rod surface and then rested for 1 h at room temperature in darkness. After this, sensors were submerged into 5 mM MCH for at least 12 h (overnight) at 4C to form the blocking layer surrounding the aptamers. 

### 2.4. Electronics and Software

The microcontroller-based potentiostat device (‘Cassio board’) used in data collection was custom designed and fabricated by Eccrine Systems, Inc. (Cincinnati, OH, USA) and is unpublished. This board uses an ADS8887DGSR 18 bit analog-to-digital converter (ADC), MAX5138BGTE+T 16 bit digital-to-analog converter (DAC), and ATSAMD21J18A-MUT microprocessor all of which can operate well above 120 Hz used for square-wave voltammetry [19,20,21]. Additional Faraday shielding was required on the board and connected cabling to use this device. Otherwise, common electromagnetic interference was found to alter measurement values. This software controlling the Cassio boards was programmed in C++ and the device is Bluetooth enabled for remote control and data reporting. While this board and software were a convenient tool for this paper, we do not believe they are specifically required for demonstrating the auto-peak-tracking results described in this paper. Relevant software code functions and segments can be found in the Supplementary Materials and should enable others to replicate this work with a variety of other programmable potentiostat devices.

### 2.5. Data Generation and Analysis

Data were collected using either the Cassio board potentiostat or a CHI684 potentiostat dependent on the specific experiment. The experiment Results section explicitly states which potentiostat, solution, and window parameters were used for each test. Common Square wave voltammetry characteristics for all tests were 120 Hz, with 1 mV steps and an amplitude of 25 mV. Signal was calculated as the difference between the peak current and average baseline window values. Signal gain was calculated independently for each sensor as the percent change from initial current differences. All data were prepared in Excel for plotting.

## 3. Fundamentals of EAB Sensor Operation and Peak Tracking

Electrochemical aptamer-based (E-AB) sensors leverage an aptamer’s conformation (shape) change in response to target analyte binding to alter a redox reporters’ availability for electron transfer with a working electrode surface, thus enabling a dynamic electrical signal response based on the concentration of analyte present. The EAB sensors further include an alkyl thiolate blocking layer to reduce background current such as oxygen reduction current and to reduce fouling of the sensor surface [22]. A powerful scanning technique for EAB sensors is square wave voltammetry (SWV), which can be further adapted for calibration-free operation [7,23]. SWV uses a customizable stair step waveform to perform bidirectional scanning to rapidly measure reductive and oxidative currents over a given potential range at frequencies of ~10–1000 Hz [24]. For all the benefit that can be extracted from EAB sensors, they have inherent limitations that restrict the translation to real-world devices including potential drift, poor longevity due to monolayer desorption (aptamer and blocking layer), and significant device complexity [14].

### 3.1. Enabling Partial Scanning to Mitigate Signal Loss

**Figure 2:**
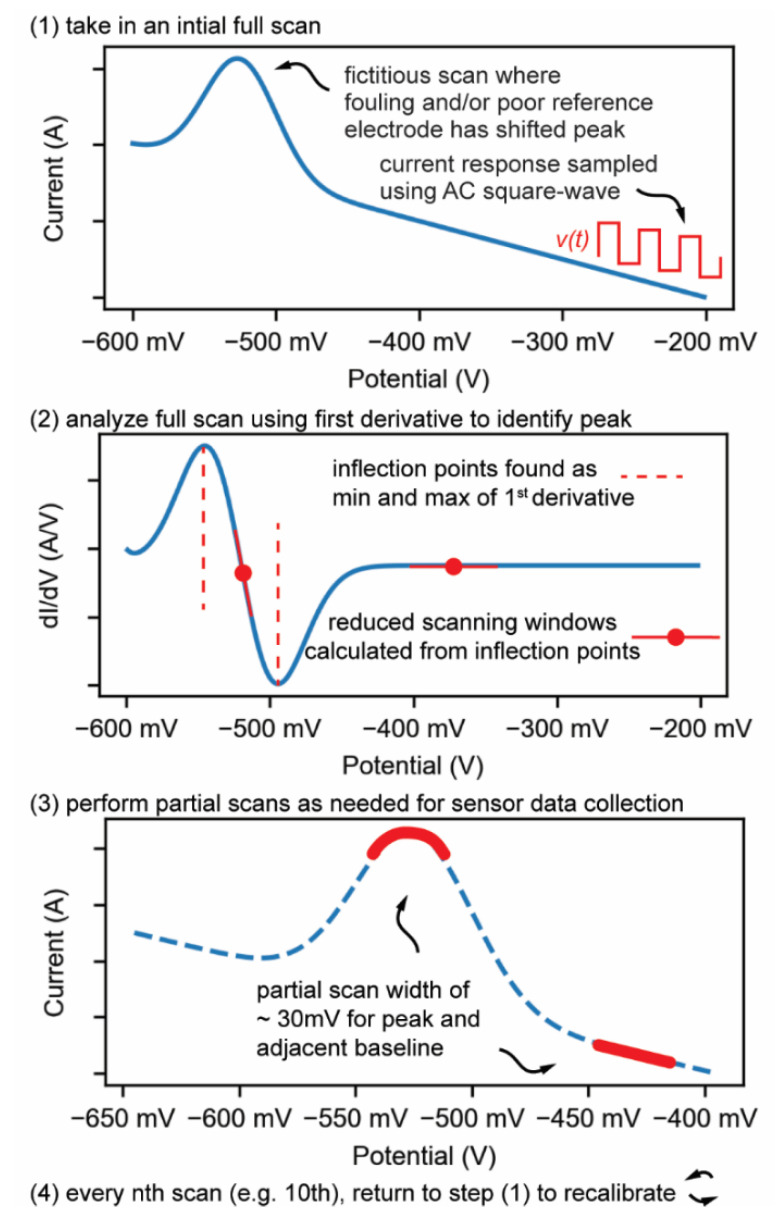
Software process for identifying peaks to generate partial scanning windows. (Step 1) Acquire voltammogram from a full scan that is not centered and is sloped. (Step 2) Take the first derivative in pursuit of the maximum and minimum values (i.e., inflection points). (Step 3) Center voltammogram peak and reduce scanning windows to baseline and peak based on inflection point potentials. (Step 4) Repeat every n scans to maintain centered peak.

A long-standing problem of E-AB scanning is that wide scanning windows of 100 s of mV’s accelerate monolayer desorption and diminish sensor signal limiting device lifetime [14]. Aptamer and blocking monolayer desorption is observable in terms of increased oxygen reduction current and signal loss [11,25]. To mitigate monolayer desorption caused by using a wide-window scanning, smart and adaptative software can be used to partially scan only the regions of SWV voltammograms used to determine redox signal which are the redox peak and baseline. For example, signal can be calculated using a 30 mV width scan of the methylene-blue redox peak and a 30 mV scan of the baseline adjacent to the peak. To scan both regions of importance with one SWV scan, a modified SWV is used that can skip irrelevant scanning regions by jumping between potentials (Figure 2). However, rapidly jumping potentials causes atypical redox currents as the distribution of methylene blue molecules that are available for oxidation or reduction is shifted compared to a traditional full-scan SWV. Therefore, the scanning potential windows for the redox peak and adjacent baseline should be small to reduce monolayer desorption but not too small as the measured redox current will be significantly affected by this oxidation/reduction population shift. 

### 3.2. Enabling Peak Tracking to Mitigate Potential Drift and to Reduce Required Electrodes

Potential drift arises from the unpredictable effects of E-AB’s environments such as fouling of working, counter, or reference electrode surfaces through the adsorption of biomolecules and the effects of salinity, pH or other solutes [17,26]. Even with an ultra-stable sealed reference electrode in a 3-electrode system meant to reduce potential drift, some potential drift and subsequent signal loss will still exist, due to fouling or monolayer desorption which alters the electrical impedance of electron transfer between the redox tag and the working electrode surface. 

To completely resolve potential drift even with a simpler two-electrode system (Figure 1d), we propose that the scanning window can simply track the redox peak as it drifts. Software accomplishes this by taking a large initial full scan to find the peak. The peak is detected by taking the first derivative of the current response with the goal of finding inflection points, maximum and minimum values of the 1st derivative (Figure 2). These inflection points are within the rising and falling edges of the voltammogram peak and scanning between the inflection points over a 30 mV range will capture the peak and exclude non-essential scanning potential ranges. These inflection points are then used as the anchor for new measurements of the peak and furthermore to partially scan a narrow 30 mV baseline current scan in subsequent scans (Figure 2). This peak-tracking process is performed every 10 scans, so the scanning edges are always updated accurately, but arguably for most testing scenarios could be performed even less frequently. If there is an abrupt and large shift in potential (change in biofluid condition, loose wire connection), a tracking algorithm could in theory lose track of the redox peak, however, the software further includes secondary failsafe functions to relocate the redox peak even if the scanning window is not accurate or the peak drifts abruptly. These failsafe functions are detailed in the Supplementary Materials (Figure S1) and are important to prevent scanning out of relevant ranges which could result in rapid electrochemical damage of the working electrode. Cumulatively, the peak tracking approach not only resolves potential drift but also permits use of a simpler 2-electrode system where the counter/reference electrode can be ex-vivo (Figure 1d).

## 4. Experimental Results

### 4.1. Partial Scanning Reduces Signal Loss

The software was first tested as individual functions and then all together, to validate that each function, both partial scanning and peak tracking, worked independently of the other and then that they also worked in combination.

The first validated function was partial SWV scanning using a CHI684 potentiostat with comparison against conventional full width SWV scanning. The full width SWV scanning window for this test was −100 to −500 mV with Ag/AgCl reference and Pt counter. The partial SWV scanning test was performed from −100 to −130 mV for baseline capture and −303 to −333 mV for redox peak capture. The input SWV waveform and output current response are provided with the Supplementary Materials (Figure S2). This test was performed in 1x PBS at 37 °C and did not involve peak-tracking.

**Figure 3:**
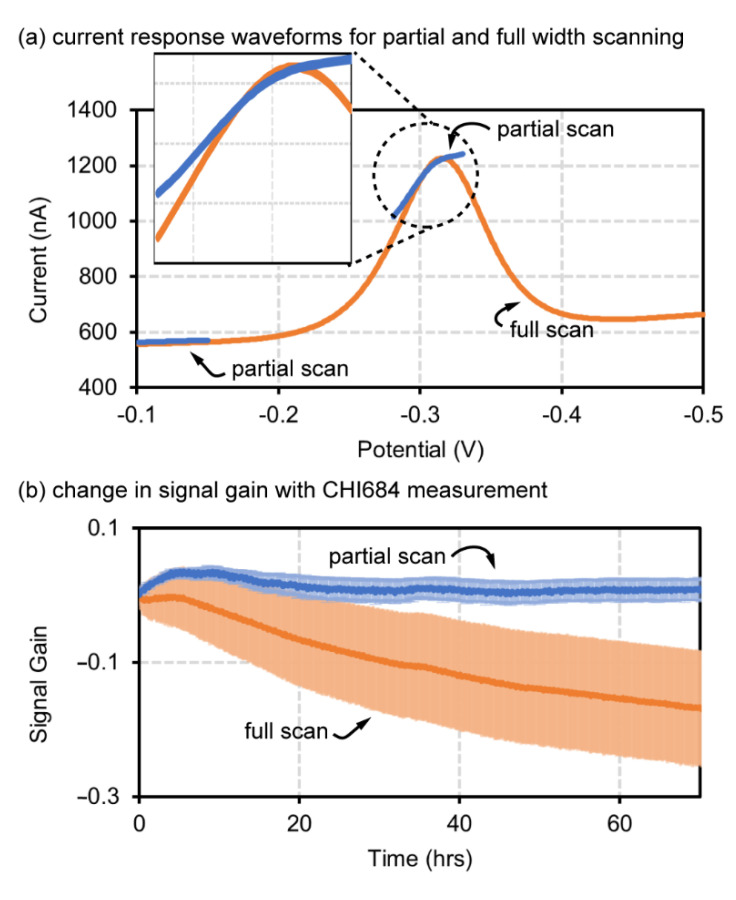
Signal gain results of partial scanning techniques. (**a**) Comparison of square wave voltammograms between conventional full scan SWV window and partial width scan SWV, with an insert comparing redox peak shape. (**b**) Signal gain degradation of full and partial scanning SWV.

Comparing full and partial scanning voltammograms (Figure 3a), the partial scanning peak has less of a parabolic shape compared to full scanning which we speculate is due to a population shift of the methylene blue redox tag oxidation vs. reduction state compared to a full scan. As shown in Figure 3b where five sensors were tested for partial and five for full scanning longevity tests, the full width scan, had ~20% loss in signal over 900 scans and 72 h while the partial scanning test maintained signal integrity and exhibited a much smaller standard deviation for each scan within the same time period (Figure 3b). The data shown in Figure 3 were obtained using the CHI684 potentiostat and were also repeated with the Cassio board potentiostat with similar results (Figure S3). 

### 4.2. Peak Tracking and Drift Protection

**Figure 4:**
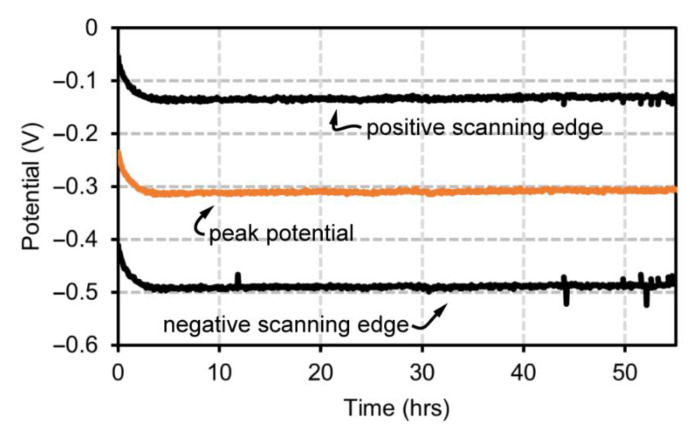
Voltage window analysis using peak tracking. Scanning edges, and peak potentials tracked with peak tracking code for over 50 h.

Next, to validate the software’s ability to track peaks and drift, a gold rod (disk) electrode was used as a pseudoreference electrode alongside a platinum counter electrode, all placed in bovine serum (Figure 4). The bare gold surface of the reference electrode is prone to rapid adsorption of proteins in serum which will significantly alter the electrical characteristics of the system causing potential drift. These tests were performed at 37 °C to mimic body conditions and hermetically sealed to prevent water loss due to evaporation. The software is expected to closely monitor the peak over the duration of any test and should not egregiously widen scanning windows into monolayer destructive voltage regions. This test did not incorporate the partial scanning function and will therefore also have loss in signal gain like in static, full scan width tests. The Cassio board potentiostat was programmed with an initial potential window of −100 to −500 mV, to be modified via software as biofouling occurs at the bare gold reference. The Cassio board was used for this test because the macro commands for the CHI684 are limited and cannot be easily modified with the peak tracking function. Over 4 days of testing and ~800 scans, the software successfully maintained the peak within the potential scanning window and the scanning window maintains similar width and relative location around the peak over the course of the experiment. The rapid initial biofouling in the first ~50 scans (2 h) is also tracked within the scanning window without failure (Figure 4).

### 4.3. Redox-Peak Tracking for a Simplified Integrated Device

**Figure 5:**
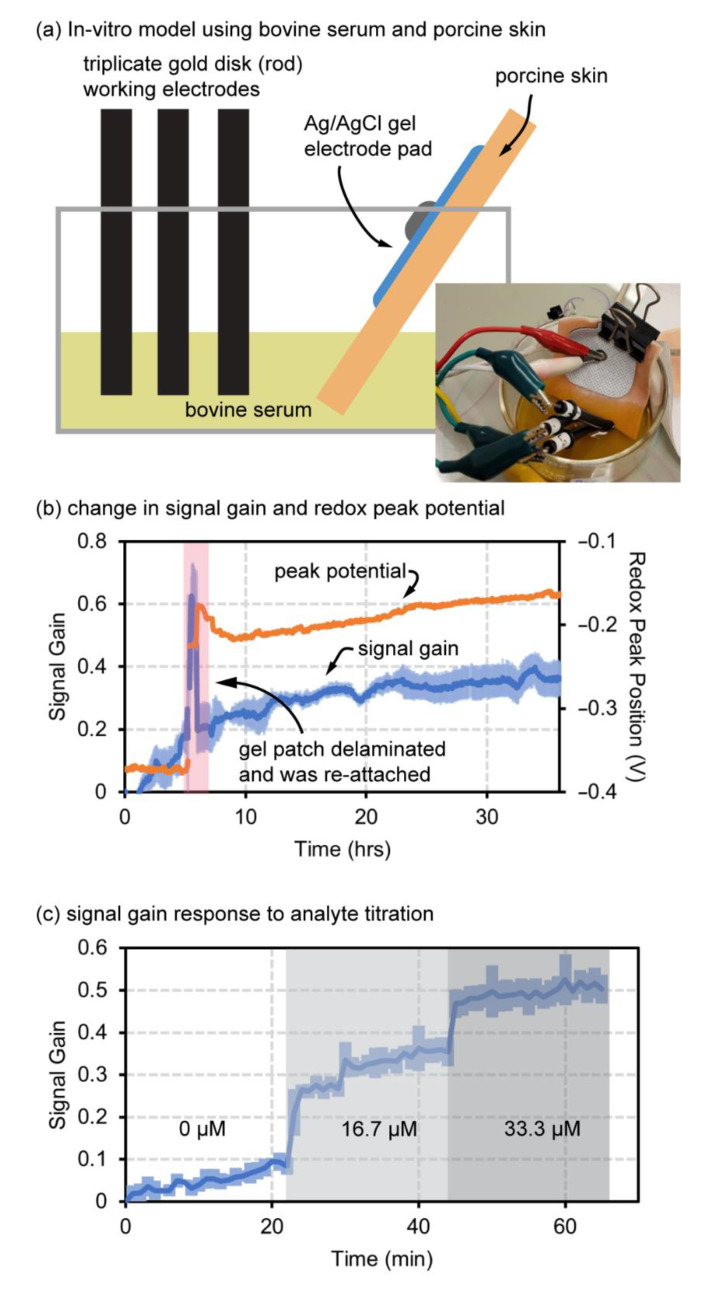
Simulated in vivo environment results. (**a**,**b**) Signal gain degradation and peak potential drift of simulated in vivo model using the peak-tracking software with porcine skin in bovine serum as a model for dermal interstitial fluid biosensing. (**c**) Signal response of peak-tracking software during vancomycin titration.

The final validation experiment was to implement an imperfect reference electrode in an unpredictable environment with both peak tracking and partial scanning enabled (Figure 5). The tests used bovine serum as the electrolyte medium, and porcine skin for placement of an Ag/AgCl electromyogram (EMG) pad electrode as a combined reference and counter electrode, as shown in Figure 5a. This simple test setup was used to mimic the 2-electrode test setup illustrated in the diagram at the right of Figure 1d. The test was unsealed and conducted at room temperature, with limited water loss causing minor changes in salinity of the serum over time. The Cassio board with enabled functions was able to both track the redox peak and prevent sensor degradation with partial scanning despite interference and imperfect conditions. The Cassio board potentiostat was provided with an initial window of −100 to −500 mV and set to obtain 30 mV windows for partial scanning of the peak and baseline. A full scan was taken every 10 scans to calculate new scanning windows. The data in Figure 5b track both signal gain and redox peak position as interpreted via the peak tracking software to continuously adjust the scanning window. At ~100 scans (~5 h) the gel electrode (counter and references) started to delaminate from the skin and was allowed to fully delaminate from the porcine skin surface and fall into the serum, and then after 5 scans (~5 min) the gel-pad electrode was then replaced onto the porcine skin. This electrode failure and replacement was permitted to challenge the robustness of the software in relocating the redox peak and continuing redox peak tracking and partial scanning windows. As shown in Figure 5b, the software recaptured a significantly shifted peak and re-established a steady signal-gain trend. This further suggests that the peak-tracking software may be able to improve the reliability and performance of wearable biosensors in ambulatory use conditions where dermal contact may vary with motion and pressure changes.

Lastly, a simple, in vitro titration was performed with the Cassio board potentiostat and software, using a Ag/AgCl reference electrode, 4 functionalized EAB sensors, and a platinum counter in 1x PBS. Titrating vancomycin presented clear regions of signal gain for each concentration demonstrating sensitivity and utility of these sensors while the scanning window is continually updated and with partial windows (Figure 5c). This final test further demonstrates that the software still works appropriately even as the redox peak height changes with addition of target.

## 5. Brief Discussion and Conclusions

In this work, a software approach for redox peak tracking is described that improves traditional SWV techniques by decreasing electrochemical degradation, corrects for any potential drift, and reduces system complexity in terms of number of required in vivo electrodes. Numerous other techniques have been demonstrated to track potential drift such as adding a second, alternative redox species at the base of an aptamer [27]. Efforts have not yet been made to reduce the required in vivo electrode set to a single working electrode but have been made to limit implantation restrictions [10]. Peak tracking can resolve all these challenges using an ultra-simple software approach without changes in sensor chemistry or device structure. While placing three electrodes in the body may not necessarily be a major challenge in cases where the number of electrodes to be placed in vivo is fixed (2, 3, 5, etc.), the software peak-tracking approach allows for a greater number of working electrodes to be placed in vivo to multiplex sensing for multiple analytes or to improve statistical accuracy with redundant sensors. Ultimately, we conclude that peak-tracking is a useful technique that not only improves longevity through partial scanning, but which can improve the in vivo reliability and adaptability of EAB sensors for eventual clinical use.

## Data Availability

All data will be made available upon request.

## Supplementary Materials

The following supporting information can be downloaded at: https://www.mdpi.com/article/10.3390/bios12100782/s1, Figure S1: Process for centering a redox peak in the scanning window, Figure S2: Input and output waveforms of partial scanning technique, Figure S3: Results from Cassio analyzer.
