# Supplementary material for: Voltammetry Peak Tracking for Longer-Lasting and Reference-Electrode-Free Electrochemical Biosensors
Source: Biosensors (Basel). 2022 Sep 22;12(10):782. doi: 10.3390/bios12100782 (PMC9599936; doi:10.3390/bios12100782)
Supplement: Supplementary file 1 [file biosensors-12-00782-s001.zip › biosensors-1892611-supplementary.pdf]

Supplementary Materials

Peak tracking code on Cassio board via inflection points:

```
void Center_Peak( int* peak_index , int max_index, int min_index, int16_t* start_volts_millivolts , int16_t* stop_volts_millivolts , int16_t step_volts_millivolts, float* velocity )
{
	// get window parameters from scan for calculation with inflection indices
	int16_t stop = *stop_volts_millivolts;
	int16_t start = *start_volts_millivolts;

	float vel;
	// prime variables for holding the inflection point indices
	int16_t min_ind = 0;
	int16_t max_ind = 0;

	int16_t len = (*(start_volts_millivolts)-*(stop_volts_millivolts));

	// Setup debounce window to avoid measuring current spike from voltage jump
	int16_t i = 5;
	float maxi = *(velocity + i);
	float mini = *(velocity + i);

	// velocity is the first derivative of the SWV voltammogram
	// run through the velocity for the max and min i.e., inflection points
	while(i<len)
	{

		vel = *(velocity+i);
		if (vel < mini)
		{
			min_ind = i;
			mini = vel;
		}
		if (vel > maxi)
		{
			max_ind = i;
			maxi = vel;
		}
		i++;
	}
	/*If the maximum index is a higher voltage magnitude than the minimum then it will flip the voltage direction. This loop will test
	the first half and second half of the window independently for the maximum and minimum to avoid flipping the signal.*/
	if (max_ind>min_ind)
```

```
	{
		i = 15;
		while(i<(len/2))
		{
			vel = *(velocity+i);
			if (vel > maxi)
			{
				max_ind = i;
				maxi = vel;
			}
			i++;
		}
		while(i>=(len/2) && i<len)
		{
			while(i<len)
			{
				vel = *(velocity+i);
				if (vel < mini)
				{
					min_ind = i;
					mini = vel;
				}
				i++;
			}
		}
	}
	// the found indices are combined with the initial parameters for the
	// inflection point potentials
	*stop_volts_millivolts = start - min_ind - WINDOW_EDGE_MILLIVOLTS;
	*start_volts_millivolts = start - max_ind + WINDOW_EDGE_MILLIVOLTS;
}
```

Failsafe for loss of peak using quadrant weight:

If the peak drifts too rapidly for the code to keep up or there is no peak within the initial scanning window, there is a failsafe built into the peak tracking code to find the peak. This failsafe uses the most recent voltammogram with poorly centered peak or absent peak and divides the voltage range into 4 quadrants. These quadrants are equally sized voltage windows and are numbered 0 to 3. The current values for each quadrant are averaged and the corresponding quadrants are given a single average current value. The code looks for the highest average quadrant values as this will likely be the rising edge of the redox peak. If a most positive quadrant can be identified, the software intelligently switches to the most negative quadrant. The scanning window will then be adjusted by an arbitrary mV value in the direction of the highest magnitude quadrant until the peak is centered. The peak is confirmed to be centered if the highest magnitude quadrant is number 1 or 2, the center 2 quadrants of the scanning window. This process is limited to 10 attempts before the code stops to prevent overvoltage and sensor degradation.

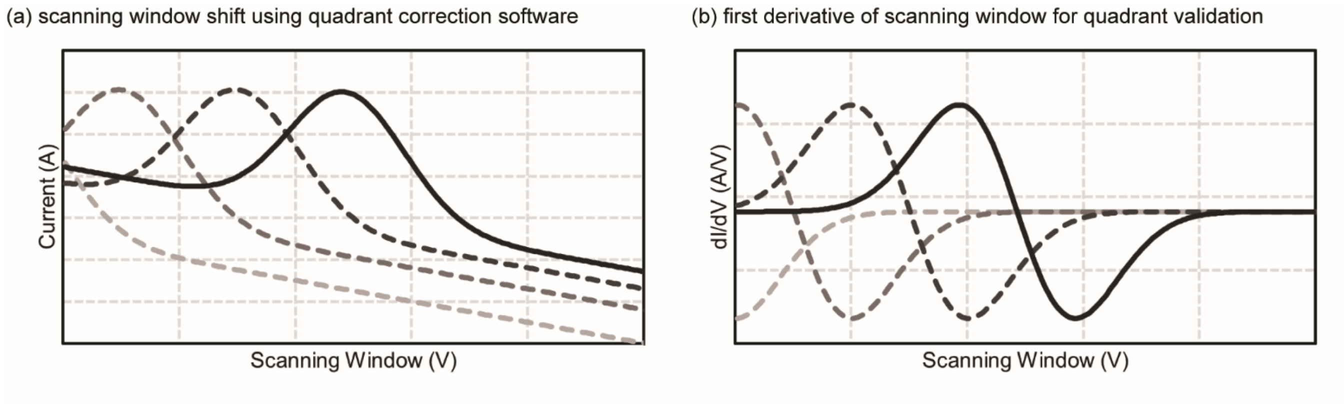

Figure S1. Process for centering a redox peak in the scanning window.

SWV waveforms taken as the scanning window shifts to center the peak. The initial waveform is offset to the left of the scanning window (light gray) and is gradually brought to center by quadrant correction (black). (b) First derivatives of the SWV waveforms used to center the peak based on the location of the falling edge of the SWV waveform. The peak is validated to be centered due to the rising edge being in the middle quadrants (black).

Peak tracking derived partial scanning EAB SWV input waveform:

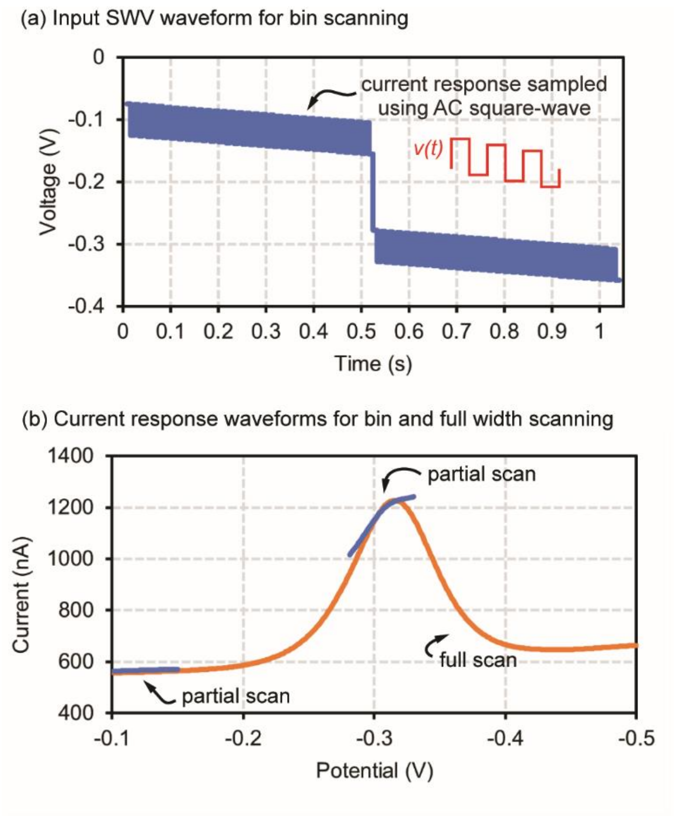

Figure S2. Input and output waveforms of partial scanning technique.

(a) Input voltage waveform used for CHI684 partial scanning including both scanning regions and voltage jump between regions. (b) Resultant output current waveforms of partial scanning and full width scanning methods.

Cassio board partial scanning EAB longevity test:

The full width SWV and initial partial scanning window for this test was -100 to -500 mV with Ag/AgCl reference and Pt counter incubated at 37ºC. Full width scanning resulted in ~10% signal loss while the partial scanning and peak tracking test gained ~20% in signal (Figure S3). The Cassio boards have higher electronic noise compared to the CHI Potentiostat but the sensors results for signal gain vs time are similar in that a partial scan is superior to a full scan.

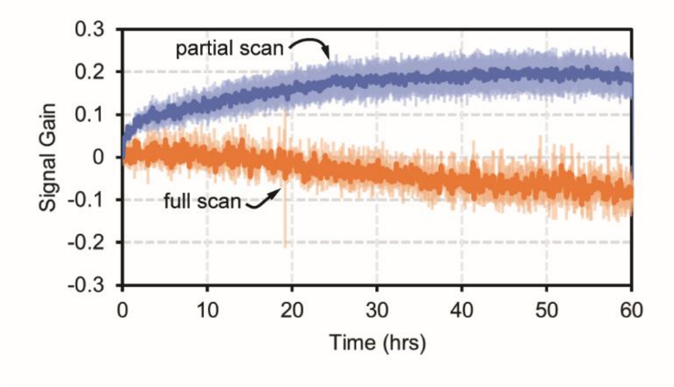

Figure S3. Results from Cassio analyzer.

Signal gain of partial scanning (blue) and full width scanning (orange) methods during a longevity test over several days.
